# Unveiling the Roles of Cysteine Proteinases F and W: From Structure to Pathological Implications and Therapeutic Targets

**DOI:** 10.3390/cells13110917

**Published:** 2024-05-25

**Authors:** Kristina Zdravkova, Olja Mijanovic, Ana Brankovic, Polina M. Ilicheva, Aleksandra Jakovleva, Jelena Karanovic, Milena Pualic, Dusan Pualic, Aleksandr A. Rubel, Lyudmila V. Savvateeva, Alessandro Parodi, Andrey A. Zamyatnin

**Affiliations:** 1 AD Alkaloid Skopje, Boulevard Alexander the Great 12, 1000 Skopje, North Macedonia; jakovlevak@hotmail.com; 2 Dia-M, LCC, 7 b.3 Magadanskaya Str., 129345 Moscow, Russia; olja.mijanovic@gmail.com; 3 Department of Forensic Sciences, Faculty of Forensic Sciences and Engineering, University of Criminal Investigation and Police Studies, Cara Dusana 196, 11000 Belgrade, Serbia; ana.brankovic@kpu.edu.rs; 4 Institute of Chemistry, Saratov State University, Astrakhanskaya Street 83, 410012 Saratov, Russia; ilichevapm@gmail.com; 5 HoxLife Science GmbH, Gutleutstraße 169-171, 60327 Frankfurt am Main, Germany; aleksandrajakovleva@hotmail.com; 6 Laboratory for Molecular Biology, Institute of Molecular Genetics and Genetic Engineering, University of Belgrade, Vojvode Stepe 444A, 11000 Belgrade, Serbia; jelena.karanovic@imgge.bg.ac.rs; 7 Institute Cardiovascular Diseases Dedinje, Heroja Milana Tepica 1, 11000 Belgrade, Serbia; milenamijan@gmail.com; 8 Military Medical Academy, Crnotravska 17, 11000 Belgrade, Serbia; pualic.d@gmail.com; 9 Laboratory of Amyloid Biology, St. Petersburg State University, 199034 St. Petersburg, Russia; a.rubel@spbu.ru; 10 Institute of Translational Medicine and Biotechnology, Sechenov First Moscow State Medical University, 119991 Moscow, Russia; ludmilaslv@yandex.ru; 11 Research Center for Translational Medicine, Sirius University of Science and Technology, 354340 Sochi, Russia; aparodi.sechenovuniversity@gmail.com; 12 Faculty of Bioengineering and Bioinformatics, Lomonosov Moscow State University, 119234 Moscow, Russia; 13 Belozersky Institute of Physico-Chemical Biology, Lomonosov Moscow State University, 119992 Moscow, Russia

**Keywords:** cysteine cathepsins, pathology, therapy, inhibitors

## Abstract

Cysteine cathepsins F and W are members of the papain-like cysteine protease family, which have distinct structural features and functional roles in various physiological and pathological processes. This review provides a comprehensive overview of the current understanding of the structure, biological functions, and pathological implications of cathepsins F and W. Beginning with an introduction to these proteases, we delve into their structural characteristics and elucidate their unique features that dictate their enzymatic activities and substrate specificity. We also explore the intricate involvement of cathepsins F and W in malignancies, highlighting their role as potential biomarkers and therapeutic targets in cancer progression. Furthermore, we discuss the emerging roles of these enzymes in immune response modulation and neurological disorders, shedding light on their implications in autoimmune and neurodegenerative diseases. Finally, we review the landscape of inhibitors targeting these proteases, highlighting their therapeutic potential and challenges in clinical translation. This review brings together the diverse facets of cysteine cathepsins F and W, providing insights into their roles in health and disease and guiding future investigations for therapeutic advances.

## 1. Introduction

Cathepsins are pivotal components of the lysosomal proteinase system [1] and play a key role in several cellular processes that are critical for maintaining homeostasis. Cathepsins are commonly classified as cysteine, serine and aspartic proteases according to the amino acid included in their active site. Among these, cysteine cathepsins F (CTSF) and W (CTSW) represent a novel subfamily that has captivated researchers with their intriguing properties and emerging importance in pathological conditions. CTSF and CTSW are both cysteine proteinases, evolutionarily distinct from other human cysteine cathepsins, and their genes are characterized by a highly conserved organization and chromosomal localization. The CTSF gene, also known as CTSF/CLN13, is located on Ch11q13, as is CTSW, highlighting their close association (Ch11q13.1) [2].

This remarkable conservation suggests a high probability that inhibitors and enhancers identified in one species can be successfully translated across different species [3]. According to phylogenetic analysis, the presence of the “ERFNAQ” motif in the propeptides of CTSF and CTSW, as well as the highly conserved genomic organization and chromosomal localization of their genes, led Wex et al. to identify CTSF and CTSW as members of a novel subset of cathepsin proteases known as “cathepsin F-like” proteases. These cysteine proteases are evolutionarily distinct from other human cysteine proteases and are closely related, with CTSF sharing 42% identity (58% homology) with CTSW. The ERFNAQ motif is also present in the mouse homologs of the CTSF and CTSW genes and in two invertebrate cysteine proteases, highlighting its evolutionary conservation (Table 1) [4].

However, both enzymes share a relatively low degree of protein sequence identity with other cysteine proteases (20–30%) [5,6]. 

More importantly, CTSF and CTSW play a unique role in cancer, exhibiting both anti-cancer and differentiation properties. They have been shown to suppress tumor growth by inhibiting angiogenesis, cell proliferation and invasion, suggesting their potential as therapeutic targets for cancer treatment. On the other hand, CTSF and CTSW also play a crucial role in orchestrating the immune system by modulating immune cell activation and effector function. They have been implicated in the regulation of dendritic cell maturation, T-cell activation and macrophage polarization, highlighting their importance in immune system homeostasis.

Given their unique properties and diverse roles in pathological conditions, CTSF and CTSW have emerged as compelling targets for therapeutic intervention and disease management. Further investigation into their molecular mechanisms and regulation is warranted to unlock their full potential for therapeutic applications. 

## 2. Structure of Cysteine Proteinases F and W

CTSF is not composed of a single sequence comprising a propeptide and a catalytically mature active region. Instead, it features an additional N-terminal segment with notable structural resemblance to the functional domains of cysteine protease inhibitors [7,8,9]. The CTSF gene encodes a polypeptide with a molecular weight of approximately 53 kDa, consisting of 484 amino acids with five N-glycosylation sites. In 1999, Santamaria et al. [9] described the complete nucleotide sequence of the CTSF cDNA, its chromosomal location and its expression profile in various human tissues and cancer cell lines. To identify and characterize this protease, they analyzed the human EST databases and compared the sequence of this enzyme with sequences of previously solved human cysteine proteinases. The EST analysis revealed that the highest degree of similarity was found with a cysteine proteinase from *Schistosoma mansoni* (48%). Significant similarities were also found with the various human cysteine proteinases, ranging from 37% with CTSL2 to 26% with CTSB. The amino acid sequence alignment of all known cysteine proteinases allowed the identification of conserved residues that are also present in CTSF and CTSW (e.g., alanine residue at position 271, proline residue at position 12, active site Cys residue, His-431 and Asn-451 residues, Trp residue and Gln residue at position 289) [10]. 

The study by Santamaria et al. [9] showed that the C-terminal region contained conserved aromatic and Gly residues flanking the active site His and Asp residues, emphasizing the relationship of CTSF to the already known cysteine proteinases [9]. The electrostatic potential of the CTSF cleft, calculated using MOLCAD software, is electronegative or neutral. According to the self-consistency evaluation obtained by PROCHECK, the model of CTSF has three disulfide bonds, which are conserved among cysteine proteases [11]. Cathepsin F, similar to other cysteine proteinases, is delivered to the endosomal/lysosomal compartment through glycosylation of at least one N-site on the protein. This glycosylation is recognized by the mannose-6-phosphate (M6P) receptor, which mediates targeting to lysosomes [12,13]. 

The CTSW cDNA encodes a putative polypeptide of 376 amino acids with a calculated molecular weight of approximately 42 kDa [14]. There are 11 exons in the *CTSW* gene, 10 of which encode cysteine proteinases [14]. CTSW contains all the highly conserved residues found in papain-like enzymes, including the residues of the catalytic triad (Cys-153, His-291, Asn-330), Gln19 of the putative oxyanion hole, Trp177 in the S1′-binding pocket, and Gly67 and Gly68 (papain numbering) (Table 2). Compared to papain, the Gly-23→Cys substitution of CTSW may lead to a higher degree of peptide bond cleavage selectivity [5,6]. 

Meinhardt et al. discovered new proteoforms of CTSW with altered amino acid sequence of the protein. Specifically, a variant resulting in the deletion of exon 6 causes a premature stop codon, leading to the production of a truncated and non-functional protein.

Notably, the C-termini of the novel proteoforms are shorter than those of wild-type CTSW. Intron 10-containing proteoforms are more abundant, suggesting their physiological relevance. Since wild-type CTSW differs from all other cysteine proteinases in its C-terminal extension, Meinhardt et al. [15] hypothesized that the C-termini of the different CTSW proteoforms may play a regulatory role in the subcellular localization of this enzyme. The wild-type variant is the only one found in natural killer (NK) cells, while the intron 10-containing isoform has also been found in gastric mucosal intraepithelial lymphocytes and has been suggested to be involved in gastro-esophageal reflux disease, but no association has been found [16].

**Figure 1:**
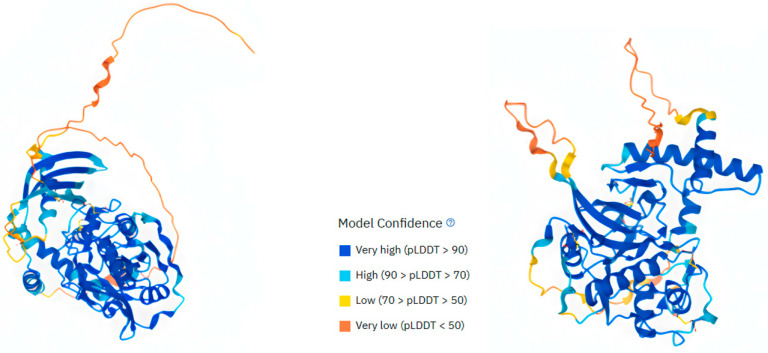
CTSF and W structure. Predicted (AlphaFold) 3D structures of CTSF (**left**) and CTSW (**right**). AlphaFold produces a per-residue model confidence score (pLDDT) between 0 and 100. Some regions below 50 pLDDT may be unstructured in isolation (source: https://www.genecards.org/cgibin/carddisp.pl?gene=CTSF, https://www.genecards.org/cgi-bin/carddisp.pl?gene=CTSW, both accessed on 3 May 2024).

Although the structures of both enzymes have not yet been solved, predicted (AlphaFold) 3D structures obtained from the Gene Card site are shown in Figure 1.

CD8+ T lymphocytes (cytotoxic T cells, CTLs) and natural killer (NK) cells primarily express CTSW mRNA, with interleukin-2 regulating its expression. [2,17,18,19]. In particular, quantitative RT-PCR studies showed that NK cells express approximately 21 times more CTSW transcripts than CTLs. Furthermore, in response to IL-2, *CTSW* gene and protein expression increased more significantly in NK cells (7-fold) than in cytotoxic T cells (CTLs) (2-fold), suggesting that CTSW is specifically expressed in these immune cells [20]. Wex et al. showed that decreased expression of endogenous CTSW, mediated by antisense oligonucleotides, corresponded to a downregulation of the cytotoxic activity of IL-2-dependent NK-92 cells. Thus, the results of this study confirmed the hypothesis that CTSW plays a central role in cellular cytotoxic processes mediated by NK cells [21,22]. 

In contrast to other cysteine proteinases, human and mouse CTSW is localized to the rough endoplasmic reticulum (ER), although the mechanisms by which CTSW is anchored to this compartment remain unclear [23]. CTSW was found mainly in immune system tissues. High levels of mRNA were found in the spleen, peripheral blood and lymph nodes. Moderate levels were found in the bone marrow and appendix. The lowest levels were found in the thymus [5]. 

## 3. Involvement of CTSF and CTSW in Malignancies

Among many functions, cysteine proteinases are also involved in the process of tumorigenesis through their ability to promote autophagy and degradation of the extracellular matrix by activating and degrading cytokines, chemokines and various growth factors, thereby modifying the tumor microenvironment and promoting angiogenesis [23,24,25]. The expression pattern of different cysteine proteinases differs in different tissues and between primary and metastatic tumors, further enhancing the specificity of the activity of these enzymes [26].

### 3.1. CTSF

To understand the expression pattern of CTSF in human tissues, Santamaria et al. [9] performed Northern blot analysis, which showed variable expression in most of the tissues examined. The main sites of CTSF expression were skeletal muscle and testis, while no expression of this enzyme was detected in leukocytes and the thymus. Furthermore, increased CTSF expression has been demonstrated in several cancer cell lines (e.g., HeLa cells), suggesting that this enzyme may play a role in the progression of some human malignancies [8]. This correlation has also been observed for other cysteine proteinases whose activity has been associated with neoplastic events [27]. 

Hybridization of Northern blots containing poly(A)1 RNA extracted from different cancer cell lines with full-length CTSF cDNA showed that the transcript in normal tissue is identical in size to that observed in HeLa cells, but the number of transcripts is increased. Reduced transcript levels were detected in melanoma, K-562 and lung carcinoma cells [9]. 

While recent studies have shown increased expression of CTSF in various cancer cell lines, the exact role of this enzyme in tumor initiation, progression and prognosis remains elusive. In the kidney cancer cell lines 769-P and A-498, more than 88% of CTSF was localized in the nucleus. However, despite extensive research aimed at elucidating this phenomenon, no definitive consensus has been reached regarding its molecular functions within the nucleus [28].

A qualitative proteomics study by Wei et al. in 2022 [29] identified elevated levels of CTSF and other candidate proteins, including fibulin-1, in brain metastatic lesions from non-small cell lung cancer (NSCLC) patients with brain metastases (BMs) compared to NSCLC tissues without BMs and primary brain tumor samples. This study aimed to identify molecular biomarkers for the early diagnosis and assessment of NSCLC. This approach might help in distinguishing between NSCLC patients with and without BMs, keeping track of disease progression, and improving patient survival [29]. On the other hand, CTSF expression continued to decrease with advancing cancer stages and metastasis, while increased CTSF expression was detected in tissues of patients with higher overall survival, highlighting the role of CTSF in attenuating NSCLC development [30]. It is likely that previous research [9] did not consider the stage of the disease or the presence of metastases, which explains the discrepancies with the newly reported data [29,30]. In addition, the first study did not define the type of lung cancer studied [9].

The bioinformatics study by Song et al. [30] showed that CTSF is downregulated in NSCLC tissues compared to healthy tissues. Gene–gene interaction analysis showed that CTSF activity is associated with genes involved in immune responses, and immunostaining showed higher expression of CTSF in macrophages and other infiltrating immune cells compared to tumor cells. In addition, 10 proteins belonging to HLA class II and MHC were shown to interact with CTSF, confirming its role in the regulation of immune responses [28]. In this context, GeneMANIA analysis revealed a correlation between CTSF and CTSW within a group of 20 related genes. This correlation encompasses their endopeptidase activity, cellular and tissue localization, and interaction with MHC class II [30]. As previously shown, CTSF is not detected in lymphocytes or the thymus [27]. For this reason, we can speculate that CTSF may play a more prominent role in the humoral immune response compared to the cellular immune response.

Thyroid cancer manifests as a distinct type of malignancy characterized by significant expression of CTSF. This association has been confirmed by research conducted by Wang et al. [31] using whole-genome sequencing (WGS) of individuals diagnosed with papillary thyroid cancer (PTC), the most common thyroid cancer resulting from a combination of environmental and genetic factors [32]. The results revealed 127 alterations (genetic changes) shared by at least two members within the families analyzed, distributed across 125 genes [31]. Among the pathogenic variants, a variant in the *CTSF* gene characterized by C>T at position 66,332,107 in the cytoband 11q13.2 was also identified. There were also more distant variants: G>A 1243 bp of the cDNA and an amino acid variant from Gly to Arg at the amino acid number 415 of this transcript. It is worth mentioning that an increased expression of CTSF compared to the wild type was observed as a result of the gene variation. As most of the family members had C>T in rs200426008, immunohistochemical confirmation was required. This analysis revealed significantly increased immunoreactivity of the mutated *CTSF* gene in PTC tissue compared to healthy tissue, highlighting the potential role of *CTSF* variations as a predictive factor for PTC inheritance and thyroid cancer inheritance in general [31].

Recently, it has been suggested that a panel of serum biomarkers including CTSF could serve as a prognostic and diagnostic tool to predict survival in gastric cancer (GC) patients. The panel showed high diagnostic performance with 95% specificity and 92% sensitivity in discriminating GC patients from healthy individuals [33]. The results showed that CTSF mRNA expression was lower in the malignant tissues than in the normal tissues. Ji et al. demonstrated that the downregulation of CTSF expression favored the proliferation of GC cells and inhibited apoptosis [18]. Furthermore, downregulation of CTSF expression in GC tissues was associated with tumor invasion, differentiation and lymph node metastasis. To investigate the role of CTSF in GC, they silenced CTSF expression using Lenti-shRNA and showed significantly increased proliferation and decreased apoptosis in the GC cell lines [18]. On the other hand, Zheng et al. demonstrated the suppressive function of CTSF on the proliferation, invasion and migration of GC cells by upregulating the *CTSF* gene via the long non-coding RNA-LINC00982. In this case, the InkRNA-LINC00982 targeted the expression of HEY1 (transcription factor belonging to the basic helix–loop–helix–orange family of transcription factors), which has been shown to be an indicator of poor clinical outcome in several cancers, such as pancreatic, colorectal, esophageal and thyroid cancers. HEY1 is associated with the downregulation of the CTSF gene, confirming an inverse association between CTSF expression and GC proliferation [34].

The drug Eltrombopag (EO) effectively suppresses the expression of lysosomal autophagy genes at the transcriptional level. In addition, EO increases the sensitivity of glioblastomas to temozolomide treatment both in vitro and in vivo. Specifically, EO impairs autophagy and reduces the protein levels of TFEB targets such as LAMP1, CTSF and HEXA genes in tumors derived from a glioblastoma xenograft mouse model. When EO is co-administered with temozolomide in glioblastoma xenograft mice, tumor proliferation rates are significantly reduced and survival is prolonged [34]. According to previously published works [28,29], the expression level of CTSF changes depending on the stage of the malignancy; therefore, the benefits of this drug should be studied in patients with different stages of cancer. 

### 3.2. CTSW

Analysis of The Cancer Genome Atlas (TCGA) database projections for patients with uterine corpus endometrial carcinoma (UCEC) showed that tumor cells express CTSW at significantly lower levels than normal cells. This hypothesis was further supported by qRT-PCR studies, which showed that primary cells expressed CTSW at higher levels than HEC-1A and Ishikawa cells. Patients with different risk factors based on novel immune risk score (NIRS) showed the same expression pattern [35]. 

In most cancers, elevated levels of *CTSW* gene expression correlate with a favorable prognosis for cancer patients. However, a patient’s ancestry may impact the expression of this gene. A multi-cancer study by Lee et al. found that CTSW expression was significantly lower in African patients compared to European patients with head and neck squamous cell carcinoma (HNSCC). This disparity was linked to a higher relative risk of mortality from the disease [28]. 

Increased expression of CTSW has been positively correlated with survival in breast cancer patients and is thought to enhance the immune response against early cancer cells. Zhang et al. demonstrated that the expression of CTSW in tumor-infiltrating lymphocytes (TILs) may be influenced by the breast cancer-associated variant rs3903072. Their findings indicate that, unlike other quantitative trait locus (QTL) genes where elevated expression typically promotes cancer progression, increased CTSW expression in TILs may enhance the cytotoxic activities of immune cells, boosting their capacity to eliminate cancer cells [35]. This research demonstrated the possibility that a cancer-associated genetic variant may regulate a gene not only in the primary tumor (parenchyma) but also in the surrounding supportive tissue (stroma), influencing the immune system’s surveillance of T lymphocytes and natural killer cells and ultimately affecting the elimination of early cancer-initiating cells [35]. In a study of invasive breast cancer (BC) patients with BRCA variants, the expression levels of five metastasis-specific proteins (CTSW, MRS2, SDCB2, RTN4 and RAD23B) were found to correlate with increased overall survival. This intriguing observation suggests that these proteins could serve as potential exosome markers for BC metastasis. Notably, three metastatic BC cell lines, particularly those with lung metastases, showed significantly reduced levels of CTSW in their exosomes. Furthermore, lower levels of CTSW in exosomes were associated with reduced gene expression and poorer survival in invasive BC patients [36].

Uterine corpus endometrial carcinoma (UCEC), a common cancer in women worldwide, exhibits considerable heterogeneity, leading to variable prognostic outcomes. To address this variability, The Cancer Genome Atlas (TCGA) database was used to obtain gene expression profiles of UCEC samples. A survival risk score was derived using multivariate Cox proportional hazards regression and a six-gene signature, including *CTSW*, to assess the likelihood of patient survival. The formula for calculating the risk score is risk score = (−0.4377) × PCSK4 level + (−0.5322) × IHH level + 0.4211 × CTSW level + (−0.3115) × LRRC8D level + (−0.0673) × TNFRSF18 level + 0.1499 × CDKN2A level. This signature was further validated in an independent test dataset and across the entire TCGA dataset, demonstrating its robust predictive ability. These findings pave the way for the identification of novel prognostic biomarkers and the development of tailored treatment strategies for UCEC patients [37]. According to the findings of Zhang et al., CTSW, together with CD3D and CD48, can be used as predictive markers for UCEC prognosis and responsiveness to immunotherapy, as they discovered a significant positive association between their expression levels in this disease. The coordinated expression of CTSW, CD3D and CD48 in UCEC could be due to their coregulation by common transcription factors or regulatory elements, or their involvement in overlapping signaling pathways or regulatory networks. This coordinated expression suggests a functional interplay between these molecules in immune activation. However, it is still unknown how CTSW modulates immunity in UCEC, in particular how it affects CD8+ T-cell expression. Gene set variation analysis revealed several significant changes in immune pathways between the high and low new immunological risk score groups, suggesting the need for further research [38].

According to the study by Pan et al., CTSW plays an important role in the development of colorectal cancer (CRC) and may serve as a potential therapeutic target. Their results indicate that both CTSW and FABP4 are positively associated with the immune response (including the cGAS-STING pathway) and DNA damage repair processes. However, while FABP4 is positively correlated with genes involved in epithelial–mesenchymal transition (EMT), CTSW shows an inverse relationship with N-cadherin and MMP9 expression, suggesting that it may suppress cell migration. These findings highlight the critical role of CTSW and FABP4 in CRC metastasis and immune response [39].

The grade, stage and invasiveness of endometrial cancer depend on the presence of specific immune cell populations. One study found positive correlations between CTSW mRNA expression in tumors and levels of infiltrating B cells, CD8+ T cells, CD4+ T cells, macrophages and dendritic cells. Functional enrichment analysis revealed that these genes were primarily associated with T-cell activation and response. In addition, Kaplan–Meier survival analysis showed a strong association between overall survival in EC patients and the expression of specific genes, including TMEM150B, CACNA2D2, TRPM5, NOL4, CTSW and SIGLEC1. These findings suggest that the tumor microenvironment has a significant impact on the clinical outcome of EC patients. This research holds promise for the development of new prognostic biomarkers and immunotherapies for EC [40]. According to Chen et al., CTSW may influence EC growth by affecting T-cell biology. CTSW and SIGLEC1 were found to be positively correlated with overall survival in EC patients. This suggests that these proteins may be potential targets for immunotherapy in EC patients [40]. The predictive and diagnostic use of CTSW in pancreatic ductal adenocarcinoma (PDAC) was suggested by data presented by Khojasteh-Leylakoohi et al. The results of the survival study showed that PDAC patients had a reduced survival rate due to overexpression of hsa.miR.153.1, hsa.miR.539 and hsa.miR.412 and decreased expression of hsa.miR.642a, hsa.miR.363, CD22, BTNL9 and CTSW [41]. Studies in dogs, particularly those with epitheliotropic lymphoma (a type of cutaneous T-cell lymphoma), have identified microarray probes for CTSW, TRAT1 and KLRK1 as potential tools to differentiate this lymphoma from other forms of interface dermatitis. These findings provide the veterinary community with much-needed biomarkers for improved diagnosis [42].

Based on the results of the aforementioned studies, it can be suggested that increased CTSW expression is positively associated with enhanced cellular immune response and serves as a biomarker of increased survival rate.

The pan-cancer study by Lee et al [43] which included 9818 cancer patients, shows significant differences between racial and ethnic populations based on cancer survival disparities (CSDs), genetic background/ancestry (GA) and tumor molecular signatures across 33 cancers. While GA correlated with race, the same parameter showed marked differences in CSD for four cancers: breast invasive carcinoma (BRCA), head and neck squamous cell carcinoma (HNSCC), renal clear cell carcinoma (KIRC) and skin cutaneous melanoma (SKCM). Moreover, CTSW emerged as one of the ancestry-related genes with significantly elevated expression, correlating with worse clinical disease status. This enzyme, which is underexpressed in individuals of African ancestry compared to European ancestry, was linked to an increased relative risk of mortality in head and neck squamous cell carcinoma (HNSCC) patients of African descent [43].

In conclusion, the expression of CTSF and CTSW is highly variable in human tissues and, in some cases and compared to other cysteine proteinases, is associated with antitumor properties and immune system biology. Understanding the role of CTSF and CTSW in tumor initiation and progression is essential for the development of novel prognostic biomarkers and immunotherapies. 

## 4. Involvement of CTSF and CTSW in Immune Response and Neurological Conditions

### 4.1. Immune Response

The function of cysteine proteinases is closely related to their role in immune cell activity. Together with their endogenous inhibitors, cysteine proteinases participate in proteolytic reactions that mediate the interplay between immune and cancer cells [44].

**Figure 2:**
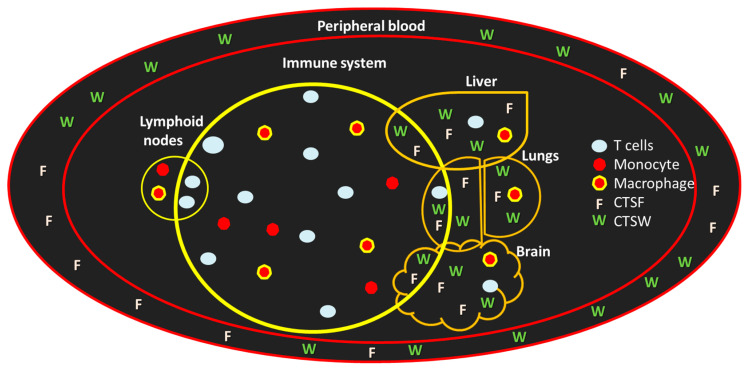
Expression of CTSF and W in the immune system. CTSF is expressed in monocyte-derived macrophages. CTSW is located in T cells in the immune tissue, with high levels of mRNA found in the spleen, peripheral blood and lymph nodes, moderate levels in the bone marrow and appendix, and the lowest levels in the thymus. Both CTSW and CTSF can circulate in different tissues through the peripheral bloodstream. The schematics represent the cells and the organs in which CTSF and CTSW are expressed.

Evidence indicates that cysteine proteinases, particularly CTSF, are implicated in immune responses through their expression in monocyte-derived macrophages [45,46] (Figure 2). CTSF has been shown to contribute to atherogenesis by remodeling the extracellular matrix and is upregulated by angiotensin II. CTSF is overexpressed in monocyte-derived macrophages within atherosclerotic lesions [47,48,49].

A study by Shi et al. highlighted the role of CTSF in degrading the MHC class II-associated invariant chain (Ii), essential for the function of MHC class II molecules in macrophages. While cysteine proteinases L and S perform this function in the thymus and lymphoid organs, macrophages deficient in cysteine proteinases L and S can still process Ii and load peptides onto MHC class II due to CTSF’s involvement in generating CLIP (class II-associated invariant chain peptide) from li-MHC class II complexes, thereby mediating MHC class II maturation and peptide loading [49].

Pires et al. demonstrated the role of cysteine proteinases in the survival of Mycobacterium tuberculosis (MTB) in human macrophages. They hypothesized that MTB affects the gene expression and proteolytic activity of cysteine proteinases B, D and S, resulting in increased expression of these cysteine proteinases activated by interferon-γ (INF-γ) macrophages (M1). However, although most cysteine proteinases have increased expression in the presence of this pathogen due to their pathogen-killing role, in the case of CTSF, its expression was significantly reduced after M1 activation, leading to increased intracellular survival of the pathogen [50]. 

Since the majority of T cells are represented by immature CD4 and CD8+ thymocytes [51], and CTSW is almost exclusively expressed in CD8+ T cells, it has been hypothesized that CTSW induction may be associated with T-cell maturation in the thymus [5]. Further research has shown that CTSW mRNA expression increases during the differentiation of thymocytes into CD8+ T cells, with concomitant inhibition of CD4 co-receptor expression [52]. 

However, the generation of a CTSW-null mouse showed that the ability of mouse CTSW-deficient CD8+ T and NK cells to induce target cell apoptosis was unaffected. Therefore, it was concluded that mouse CTSW is not involved in cell-mediated cytotoxicity, regardless of its restricted expression [14]. In addition, it was shown that CTSW knockdown using small interfering RNA (siRNA) did not affect INF-γ production by CD8+ T cells, thus excluding an essential role of CTSW in the cytotoxicity process [53].

It has been shown that CTSW-expressing cytotoxic cells may play a significant role in the pathogenesis of autoimmune gastritis compared to their involvement in other gastric diseases and inflammatory bowel diseases. The observed increase in CTSW-expressing cytotoxic cells within autoimmune gastritis compared to its decrease in celiac disease and ulcerative colitis highlights the distinct immune cell profiles involved in the pathogenesis of these gastrointestinal diseases [14,54]. According to current data, CTSW appears to act as a gatekeeper for peripheral regulatory T-cell differentiation and mucosal immune quiescence in mice. CTSW, which is significantly upregulated in pTreg cells in response to TGF stimulation, is important for the intrinsic control of pTreg cell development. pTreg cells are an important T-cell lineage for mucosal immunological tolerance and anti-inflammatory responses, and IL-2R signaling is required for pTreg cell production, growth and maintenance. CTSW deficiency results in increased pTreg cell production, which protects mice against intestinal inflammation [55].

Studies based on quantitative RT-PCR analysis of cathepsin expression in inflamed mucosa due to Helicobacter pylori infection reveal different expression patterns for different cysteine proteinases (K, L, B and W). Compared to cathepsins K, L and B, which were more widely expressed, CTSW showed lower expression levels. Each of these cysteine proteinases showed different expression patterns: CTSK (parietal cells), CTSX (macrophages), CTSL (epithelial cells) and CTSW (lymphocytes) [56]. 

The possible role and function of CTSW in influenza A virus (IAV) replication has also been investigated [56]. Knockdown of CTSW using siRNAs resulted in a reduction in the titer of IAV strains [57]. CTSW is a critical host factor for IAV entry into target cells, suggesting that it is a promising target for the development of new antiviral drugs [57,58].

A recent study–case report demonstrated the impact of certain proteins, including CTSW, in the absence of relapse in patients with chronic myeloid leukemia (CML) upon discontinuation of tyrosine kinase inhibitor (TKI) therapy. In fact, it was shown that in several cases of TKI discontinuation, an immunological mechanism is activated, leading to increased levels of NK cells, which in turn exert anti-leukemia activity. Single-cell transcriptome analyses have shown that the proteins associated with this activity are GNLY (granulysin), GZMH (granzyme H) and CTSW [59].

However, the large number of in vitro and in vivo experiments and bioinformatic analyses of the molecular mechanisms of CTSW and CTSF still do not provide a clear explanation of the role of CTSW and CTSF in the immune response to cancer.

### 4.2. Neurological Conditions 

According to quantitative RNA-sequencing data analysis from the Allen Brain Atlas database, the human brain uses well-defined and balanced patterns of cathepsin expression during different stages of its development. Overall gene expression was found to be comparable between infancy and young adulthood. Notably, the order of cathepsin expression remained consistent throughout the brain at all ages studied, with CTSB, D and F showing the highest levels of expression, CTSA, L and Z showing moderate expression, CTSC, H, K, O, S and V showing low expression and CTSE, G and W showing very low expression. Therefore, this section will be devoted exclusively to CTSF. Any imbalance in cathepsin gene expression during brain development may lead to lysosome-related brain disorders [60]. 

Variants in the *CTSF* gene were first described in mice, where CTSF-deficient mice showed symptoms of neurological disease from 12 to 16 months of age with lack of motor coordination, progressive hind limb weakness, significant weight loss and premature death [61]. Electron microscopy also showed that these mice had accumulated eosinophilic granules typical of lysosomal dysfunction in neurons, including large amounts of autofluorescent lipofuscin and pronounced gliosis, supporting the role of this gene in neurodegeneration. 

Several studies in recent years have shown a correlation between novel variants in the *CTSF* gene and neurodegenerative disorders, including dementia, early- and late-onset Alzheimer’s disease (AD), and Kufs disease type B [6,62]. Kufs disease type B is also known as adult-onset neuronal ceroid lipofuscinosis type 13 (CLN-13) and is characterized by dementia and a variety of motor symptoms [63]. Interestingly, CTSF is not the only cathepsin associated with CNL diseases—CTSD missense variants have been identified in several forms of CNL disease [64]. 

Smith et al. identified a single region on chromosome 11 where two families affected by recessive Kufs disease type B exhibited linkage. Exome sequencing of five samples from these families within this linkage region revealed homozygous and compound heterozygous missense variants in the *CTSF* gene (c.962A>G (p.Gln321Arg), c.1373G>C (p.Gly458Ala), c.1439C>T (p.Ser480Leu)) [64]. They also sequenced the *CTSF* gene in 22 unrelated individuals with suspected recessive Kufs disease and identified one patient carrying compound heterozygous variants c.962A>G (p.Tyr231Cys) and c.954delC (p.Ser319Leufs*27). The authors provided in silico functional predictions for all variants identified in this study [64]. From this study, we can speculate that *CTSF* screening could represent a marker of early-onset dementia limiting the need for invasive biopsies.

Bras et al. analyzed exomes from first-cousin family members and again identified two key variants in the *CTSF* gene that may be associated with early-onset AD. According to Polyphen-2, SIFT and VariantTaster, the first variant, 1243G>A, was homozygous and deleterious/disease-associated. The second variant, c.214-6C>T, was biallelic and heterozygous in most samples from the databases. Overall, the siblings were diagnosed with an AD phenotype with overlapping Kufs disease, likely due to the presence of biallelic variants of the *CTSF* gene [65]. 

Using exome sequencing and targeted massive parallel resequencing, a homozygous variant p.Ile404Thr in the *CTSF* gene was found to cosegregate in a Belgian family with Kufs disease, while a heterozygous variant p.Arg245His was detected in two patients with a common haplotype from a Belgian cohort of unrelated patients with frontotemporal dementia [66]. Novel compound heterozygous variants in the CTSF gene, a missense variant c.977G>T (p.C326F) and a nonsense variant c.416C>A (p.S139X), causing Kufs disease type B, have also been detected and updated with magnetic resonance imaging findings: diffuse cortical atrophy, mild hyperintensity and a reduction in deep white matter on T2-weighted images [67]. In addition, a novel homozygous frameshift pathogenic variant p.Gly439Alafs*36 in the *CTSF* gene was identified that causes Kufs disease type B while mimicking frontotemporal dementia–parkinsonism [68]. Di Fabio et al. described the neuroradiological features of Kufs disease type B in two Caucasian women carrying a homozygous c.213+1G>C variant in the CTSF gene, including brain volume reduction, periventricular and deep white matter hyperintensities, and thinning of the corpus callosum at the onset of cognitive decline, which may prompt clinicians to further investigate diagnostic signs of this disease [69]. Peters et al. demonstrated that lysosomal integral membrane protein type 2 (LIMP-2/SCARB2) is a substrate of CTSF and that Kufs disease type B-causing variants of CTSF result in its inability to cleave LIMP-2 [70].

A recent proteomic analysis of samples from children with myelin oligodendrocyte glycoprotein antibody-associated disease (MOGAD) identified potential new biomarker candidates. By comparing protein expression profiles between the MOG group and healthy controls, functional analysis revealed that the dysregulated proteins, including CTSF, were primarily involved in various biological processes such as complement and coagulation cascades, cell adhesion, axon guidance and glycosphingolipid production. However, the limited sample size in the study by Wang et al. prevented further confirmation of these potential biomarkers [71], given that previously published research shows variance in prevalence between populations [72]. 

### 4.3. Aging and Diabetes

In the process of aging—senescence—there is an accumulation of apoptotic cells and this phenomenon can lead to certain diseases such as dermatitis [73]. 

The study by Takaya et al. showed a significant upregulation of *CTSF* gene and protein expression in senescent fibroblasts and keratinocytes compared to proliferating cells, suggesting the use of CTSF as a biomarker for the detection of senescent cells. In addition, the study performed a comparative analysis of CTSF protein expression in human skin samples from individuals aged 3 and 89 years. The results showed a significant difference in the number of positive CTSF cells between the two age groups. Specifically, the younger skin tissue showed few CTSF-positive cells, whereas the older skin tissue showed a multitude of CTSF cells, confirming that this enzyme could be used as a potential biomarker for skin rejuvenation therapies targeting senescent cell removal (senolysis) [73].

Research has confirmed a causal relationship between early age-related macular degeneration (AMD) and elevated serum CTSF levels. Notably, while serum CTSF has been associated with early AMD, data analysis indicates that other cysteine proteinases did not show a significant increase in serum levels, suggesting that this protease is a notable factor involved in the pathophysiology of AMD [74]. 

In recent years, in vivo and in vitro studies have shown that adipose-derived stem cells (ADSCs) downregulate CTSF and downstream pro-apoptotic proteins (Bid, BAX and caspase 9) while increasing downstream anti-apoptotic protein expression (Bcl-2 and Bcl-XL). ADSCs protect against radiation-induced dermatitis, have an anti-apoptotic effect by suppressing CTSF expression, and may be a promising therapeutic candidate for the prevention of radiation-induced dermatitis [75].

Laser-dissected leukocyte-infiltrated and non-infiltrated pancreatic islets were analyzed for proteases and protease inhibitors by specific microarray analysis (CLIP-CHIP), quantitative real-time PCR and protein analysis. The results identified CTSS, W and C activity at the sites of leukocyte penetration of the peri-islet basement membrane in association with a macrophage subpopulation in NOD mice and human type 1 diabetic samples. This suggests that these enzymes may represent a novel therapeutic target specifically for the islet infiltration phase of type 1 diabetes [76].

Using recombinant adenovirus (rAV)-driven small hairpin RNA (rAV-sh), Saghizadeh et al. showed that silencing matrix metalloproteinase-10 (M10) and CTSF in organ-cultured diabetic corneas normalized slow wound healing while reducing the expression of markers in diabetic and stem cells. The most remarkable result was achieved when shRNAs targeting M10 and CTSF were delivered at the same time as c-met overexpression, resulting in a significant acceleration of the wound healing process compared to control corneas [77].

## 5. Inhibitors of CTSF 

The preferred protease cleavage site motif is defined as -P3-P2-P1-P1′-P2′-P3′-. Cleavage occurs between residues P1 and P1′ [78]. CTSW binds to conserved primary and non-primary sites but accepts most amino acids at the P2 position. Interestingly, when only the top 19 polypeptides recognized by TAILS are included, CTSW shows a stronger affinity for arginine (N) and lysine (K) at the P1’ and P1 positions, respectively. This property may be important in the design of highly selective CTSW inhibitors, but they are yet to be designed [57].

For this reason, only the inhibitors of CTSF are summarized in this section. CTSF is inhibited by cystatins, cysteine protease propeptides, peptidyl ketones and vinyl sulfones. Some binding constants (Ki) for CTSF inhibitors are listed in Table 2 and Table 3.

### 5.1. Endogenous Inhibitors

#### Cystatin Family

The cystatins are the largest group of endogenous cathepsin inhibitors. They are generally potent non-selective reversible inhibitors of cathepsin endopeptidases but weaker inhibitors of exopeptidases. Cystatins form a superfamily that can be divided into three major families: stefins, cystatins and kininogens [3,78,79]. Stefins (stefin A and B; also known as cystatin A and B) and cystatins are single-chain proteins that form equimolar complexes with their target enzymes. Unlike stefins, cystatins contain disulfide bonds at the carboxy terminus of the molecule and some members can be glycosylated. Kininogens are multidomain glycoproteins that can bind two cathepsin molecules with different affinities. They are composed of three cystatin-like domains, only two of which (domains 2 and 3) have cysteine protease inhibitory activity. Exosite binding inhibitors such as cystatins bind to a region adjacent to the active site, preventing substrate access to this site without directly blocking the catalytic center of the enzyme [80]. The cystatins that inhibit CTSF are listed in Table 3.

**Table 3:** Endogenous inhibitors of CTSF.

Inhibitors	Ki (nM)	Reference
Cystatin A (Stefin A)	25.000	[81]
Cystatin C	0.030	
L-kininogen	4.700	
Equistatin	0.470	
Chicken cystatin	0.060	
Cystatin F	0.170	[82]
Human p41 fragment	0.510	[83]
Propeptide of cruzipain	0.032	[84]

### 5.2. Thyropins (p41 Fragment)

Thyropins are protease inhibitors whose structures contain thyroglobulin type 1 domains. The p41 fragment sequence is homologous to these consecutive repeats and is embedded in the much larger invariant chain (p41Ii) associated with major histocompatibility complex (MHC) class II [85]. The p41 fragment inhibits human CatV, K, S and F with Ki values in the nM (nanomolar) range [86,87]. Similar to cystatins, the p41 fragment interacts with the target enzyme in a cystatin-like manner but makes more additional contacts, thereby achieving higher proteinase specificity [83].

### 5.3. Propeptide-like Inhibitors

Human CTSF was efficiently inhibited by a cruzipain propeptide (cysteine proteinase from *Trypanosoma cruzi*) [84]. The cruzipain propeptide is selective for CTSF over other mammalian cysteine proteinases. The Ki for CTSF was in the same range as that observed for the related enzyme.

### 5.4. Synthetic Inhibitors

Using the dipeptide nitrile library, Schmitz et al. evaluated their activity profile as CTSF inhibitors [88]. N-(4-phenylbenzoyl)-leucylglycine nitrile and N-(4-phenylbenzoyl)-leucylmethionine nitrile were found to be potent covalent-reversible inhibitors of human CTSF. None of the investigated dipeptide nitriles were selective for CTSF over cathepsin K, which was used as a functional reference. The structure of human CTSF with the covalent irreversible vinyl sulfone inhibitor, 4-morpholin-4-ylpiperidine-CO-Phe-Nva-VS-Ph (Morph-Pip-Phe-NvaVSPh), was determined by Somoza et al. [88,89]. A diazomethyl ketone-containing irreversible inhibitor (BIL-DMK) rapidly inactivates CTSF in isolated enzyme assays [90]. A number of commonly used small peptide caspase inhibitors such as Z-VAD-fmk (Z-ben-zyloxycarbonyl, fmk-fluoromethylketone), Z-DEVD-cmk (cmk-chloromethylke-tone) and Ac-YVAD-cmk (Ac-acetyl) efficiently inhibit CTSF [91,92] (Table 4).

To evaluate their potential anti-cancer effects and impact on the lysosomal compartment in human renal cell carcinoma, Rudziska et al. synthesized two fluoromethylketone-based peptides known for their inhibitory activity against cysteine cathepsins. The study showed that these inhibitors exhibited weaker interactions with the CTSW active site compared to other cysteine proteinases, specifically B and L. This observation was corroborated by Western blot analysis, which showed that renal cancer cells treated with these inhibitory peptides did not induce an increase in CTSW levels, in contrast to the observed effects on other cysteine proteinases [93].

## 6. Conclusions

This review aims to summarize the current state of knowledge of CTSF and CTSW biology, highlighting their distinct evolutionary pathways despite shared features such as potential anti-cancer activity and involvement in immune regulation. We conducted an extensive literature search encompassing the past 20 years, primarily using Google Scholar and PubMed databases. Despite this effort, the scarcity of recent publications on these enzymes highlights the need for further research to understand their activity and biological targets, which may reveal potential therapeutic opportunities. The identification of specific natural inhibitors and the development of new synthetic inhibitors could be key to this discovery process.

## Figures and Tables

**Table 1:** Comparison of ERFNAQ motif (**bold**) in human *CTSF* and *CTSW* genes, mouse-related homologs and ERFNAQ-related motifs in invertebrata [4].

Gene	Amino Acid Sequence
Human CTSF	E**E**ARW**R**LSV**F**VN**N**MVR**A**QKI**Q**ALDRG
Mouse CTSF	E**E**HAH**R**LDI**F**AH**N**LAQ**A**QRL**Q**EEDLG
Human CTSW	A**E**YTR**R**LSI**F**AHNLAQ**A**QRL**Q**QEDLG
Mouse CTSW	E**E**AQW**R**LTV**F**AR**N**MIR**A**QKI**Q**ALDRG
Paragonium westermani	E**D**DQK**R**FAI**F**KD**N**LVR**A**QQY**Q**TQEQG
Caenorhabditis elegans	R**E**VLK**R**FRV**F**KKNAKV**I**REL**Q**KNEQG

**Table 2:** Conserved, additional and substituted residues of CTSF and CTSW polypeptides.

**Conserved residues in CTSF**
Cys-113, His-249, and Asn-269, Gln-87, Trp-271, and Trp-275, Gly-153, Gly-154
**Additional residues in CTSF**
Cys-110, Cys-144, Cys-151, Cys-184, Cys-242, Cys-290
**Conserved residues in CTSW**
Cys-153, His-291, Asn-331, Gln-19, Trp-177, Gly-67, Gly-68
**Substituted residues in CTSW**
Tyr-67→Phe Pro-68→Val Val-133→Thr Ala-160→Ser Phe-207→Leu

**Table 4:** Synthetic inhibitors of CTSF.

Inhibitors		Ki (nM)	Reference
N-(4-phenylbenzoyl)-leucylglycine nitrile	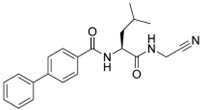	7.79	[84]
N-(4-phenylbenzoyl)-leucylmethionine nitrile	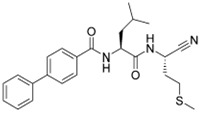	7.28	
Morph-pip-Phe-NvaVSPh	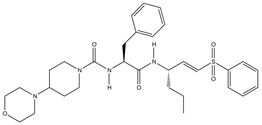		[85,86]
BIL-DMK	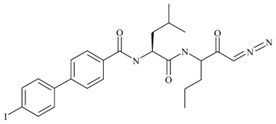		[87]
Z-DEVD-cmk			[88]
Ac-YVAD-cmk			
Z-VAD-fmk			

## References

[1] Mijanović O., Jakovleva A., Branković A., Zdravkova K., Pualic M., Belozerskaya T.A., Nikitkina A.I., Parodi A., Zamyatnin A.A. (2022). Cathepsin K in Pathological Conditions and New Therapeutic and Diagnostic Perspectives. Int. J. Mol. Sci..

[2] Wex T., Levy B., Smeekens S., Ansorge S., Desnick R., Bromme D. (1998). Genomic Structure, Chromosomal Localization, and Expression of Human Cathepsin W. Biochem. Biophys. Res. Commun..

[3] Turk V., Stoka V., Vasiljeva O., Renko M., Sun T., Turk B., Turk D. (2012). Cysteine cysteine proteinases: From structure, function and regulation to new frontiers. Biochim. Biophys. Acta BBA—Proteins Proteom..

[4] Wex T., Levy B., Wex 1., Brömme D., Langner J., Ansorge S. (2002). Human Cathepsins W and F form A New Subgroup of Cathepsins that is Evolu-tionary Separated from the Cathepsin B- and L-Like Cysteine Proteases. Cellular Peptidases in Immune Functions and Diseases 2.

[5] Linnevers C., Smeekens S., Brömme D. (1997). Human cathepsin W, a putative cysteine protease predominantly expressed in CD8^+^ T-lymphocytes. FEBS Lett..

[6] Dalton J.P., Robinson M.W., Brindley P.J., Cathepsin W. (2013). Handbook of Proteolytic Enzymes.

[7] Wex T., Levy B., Wex H., Brömme D. (1999). Human Cysteine proteinases F and W: A New Subgroup of Cysteine proteinases. Biochem. Biophys. Res. Commun..

[8] Wang B., Shi G.-P., Yao P.M., Li Z., Chapman H.A., Brömme D. (1998). Human Cathepsin F. J. Biol. Chem..

[9] Santamaría I., Velasco G., Pendás A.M., Paz A., López-Otín C. (1999). Molecular Cloning and Structural and Functional Characterization of Human Cathepsin F, a New Cysteine Proteinase of the Papain Family with a Long Propeptide Domain. J. Biol. Chem..

[10] Kamphuis I., Drenth J., Baker E. (1985). Comparative Studies Based on the High-resolution Structures of Papain and Actinidin, and on Amino Acid Sequence Information for Cysteine proteinases B and H, and Stem Bromelain. J. Mol. Biol..

[11] Fengler A., Brandt W., Langner J., Ansorge S. (2002). Development and Validation of Homology Models of Human Cysteine proteinases K, S, H, and F. Cellular Peptidases in Immune Functions and Diseases 2.

[12] Nägler D.K., Sulea T., Ménard R. (1999). Full-Length cDNA of Human Cathepsin F Predicts the Presence of a Cystatin Domain at the N-terminus of the Cysteine Protease Zymogen. Biochem. Biophys. Res. Commun..

[13] Yadati T., Houben T., Bitorina A., Shiri-Sverdlov R. (2020). The Ins and Outs of Cysteine proteinases: Physiological Function and Role in Disease Management. Cells.

[14] Buhling F., Kellner U., Guenther D., Kahl S., Brömme D., Weber E., Malfertheiner P., Wex T. (2002). Characterization of Novel Anti-Cathepsin W Antibodies and Cellular Distribution of Cathepsin W in the Gastrointestinal Tract. Biol. Chem..

[15] Meinhardt C., Peitz U., Treiber G., Wilhelmsen S., Malfertheiner P., Wex T. (2004). Identification of a novel isoform predominantly expressed in gastric tissue and a triple-base pair polymorphism of the cathepsin W gene. Biochem. Biophys. Res. Commun..

[16] Raab A.-K., Mönkemüller K., Kandulski A., Weber E., Malfertheiner P., Wex T. (2011). Expression pattern of cathepsinW-isoforms in peripheral blood and gastroesophageal mucosa of patients with gastroesophageal reflux disease. Biol. Chem..

[17] Ji C., Zhao Y., Kou Y.-W., Shao H., Guo L., Bao C.-H., Jiang B.-C., Chen X.-Y., Dai J.-W., Tong Y.-X. (2018). Cathepsin F Knockdown Induces Proliferation and Inhibits Apoptosis in Gastric Cancer Cells. Oncol. Res. Featur. Preclin. Clin. Cancer Ther..

[18] Vidak E., Javoršek U., Vizovišek M., Turk B. (2019). Cysteine Cysteine proteinases and their Extracellular Roles: Shaping the Microenvironment. Cells.

[19] Novinec M., Lenarčič B., Turk B. (2014). Cysteine Cathepsin Activity Regulation by Glycosaminoglycans. BioMed Res. Int..

[20] Wex T., Bühling F., Wex H., Günther D., Malfertheiner P., Weber E., Brömme D. (2001). Human Cathepsin W, a Cysteine Protease Predominantly Expressed in NK Cells, Is Mainly Localized in the Endoplasmic Reticulum. J. Immunol..

[21] Wex T., Wex H., Hartig R., Wilhelmsen S., Malfertheiner P. (2003). Functional involvement of cathepsin W in the cytotoxic activity of NK-92 cells. FEBS Lett..

[22] Quesnel A., Karagiannis G.S., Filippou P.S. (2020). Extracellular proteolysis in glioblastoma progression and therapeutics. Biochim. et Biophys. Acta (BBA)—Rev. Cancer.

[23] Ondr J.K., Pham C.T.N. (2004). Characterization of Murine Cathepsin W and Its Role in Cell-mediated Cytotoxicity. J. Biol. Chem..

[24] Mijanović O., Branković A., Panin A.N., Savchuk S., Timashev P., Ulasov I., Lesniak M.S. (2019). Cathepsin B: A sellsword of cancer progression. Cancer Lett..

[25] Lin Y., Yu B., Fang P., Wang J. (2023). Inhibiting autophagy before it starts. Autophagy.

[26] Dohchin A., Suzuki J ichi Seki H., Masutani M., Shiroto H., Kawakami Y. (2000). Immunostained cysteine proteinases B and L correlate with depth of invasion and different metastatic pathways in early stage gastric carcinoma. Cancer.

[27] Berquin I.M., Sloane B.F. (1995). Cysteine proteases and tumor progression. Perspect. Drug Discov. Des..

[28] Frolova A.S., Tikhomirova N.K., Kireev I.I., Zernii E.Y., Parodi A., Ivanov K.I., Zamyatnin A.A. (2023). Expression, Intracellular Localization, and Maturation of Cysteine Proteinases in Renal Embryonic and Cancer Cell Lines. Biochemistry.

[29] Wei S., Liu W., Xu M., Qin H., Liu C., Zhang R., Zhou S., Li E., Liu Z., Wang Q. (2022). Cathepsin F and Fibulin-1 as novel diagnostic biomarkers for brain metastasis of non-small cell lung cancer. Br. J. Cancer.

[30] Song L., Wang X., Cheng W., Wu Y., Liu M., Liu R., Zhang S., Xia H., Liu H., Tai X. (2021). Expression signature, prognosis value and immune characteristics of cathepsin F in non-small cell lung cancer identified by bioinformatics assessment. BMC Pulm. Med..

[31] Wang Y., Mei J., Zhang Y., He X., Zheng X., Tan J., Jia Q., Li N., Li D., Wang Y. (2022). Cathepsin F genetic variant is associated with familial papillary thyroid cancer. Am. J. Med. Sci..

[32] Kitahara C.M., Sosa J.A. (2016). The changing incidence of thyroid cancer. Nat. Rev. Endocrinol..

[33] Yang L., Wang J., Li J., Zhang H., Guo S., Yan M., Zhu Z., Lan B., Ding Y., Xu M. (2016). Identification of Serum Biomarkers for Gastric Cancer Diagnosis Using a Human Proteome Microarray. Mol. Cell. Proteom..

[34] Zheng L., Cao J., Liu L., Xu H., Chen L., Kang L., Gao L. (2021). Long noncoding RNA LINC00982 upregulates CTSF expression to inhibit gastric cancer progression via the transcription factor HEY. Am. J. Physiol. Liver Physiol..

[35] Zhang G., Yin Z., Fang J., Wu A., Chen G., Cao K. (2023). Construction of the novel immune risk scoring system related to CD8+ T cells in uterine corpus endometrial carcinoma. Cancer Cell Int..

[36] Shen S., Tu C., Shen H., Li J., Frangou C., Zhang J., Qu J. (2023). Comparative Proteomics Analysis of Exosomes Identifies Key Pathways and Protein Markers Related to Breast Cancer Metastasis. Int. J. Mol. Sci..

[37] Wang Y., Ren F., Chen P., Liu S., Song Z., Ma X. (2018). Identification of a six-gene signature with prognostic value for patients with endometrial carcinoma. Cancer Med..

[38] Zhang Y., Manjunath M., Yan J., Baur B.A., Zhang S., Roy S., Song J.S. (2019). The Cancer-Associated Genetic Variant Rs3903072 Modulates Immune Cells in the Tumor Microenvironment. Front. Genet..

[39] Pan B., Yue Y., Ding W., Sun L., Xu M., Wang S. (2023). A novel prognostic signatures based on metastasis- and immune-related gene pairs for colorectal cancer. Front. Immunol..

[40] Chen P., Yang Y., Zhang Y., Jiang S., Li X., Wan J. (2020). Identification of prognostic immune-related genes in the tumor microenvironment of endometrial cancer. Aging.

[41] Khojasteh-Leylakoohi F., Mohit R., Khalili-Tanha N., Asadnia A., Naderi H., Pourali G., Yousefli Z., Khalili-Tanha G., Khazaei M., Maftooh M. (2023). Down Regulation of Cathepsin W Is Associated with Poor Prognosis in Pancreatic Cancer. Sci. Rep..

[42] Olayinka J.T., Nagarkar A., Ma D.J., Wong N.B., Romasco A., Piedra-Mora C., Wrijil L., David C.N., Gardner H.L., Robinson N.A. (2023). Cathepsin W, T-cell receptor-associated transmembrane adapter 1, lymphotactin and killer cell lectin like receptor K1 are sensitive and specific RNA biomarkers of canine epitheliotropic lymphoma. Front. Veter-Sci..

[43] Lee K.K., Rishishwar L., Ban D., Nagar S.D., Mariño-Ramírez L., McDonald J.F., Jordan I.K. (2022). Association of Genetic Ancestry and Molecular Signatures with Cancer Survival Disparities: A Pan-Cancer Analysis. Cancer Res..

[44] Brown J., Matutes E., Singleton A., Price C., Molgaard H., Buttle D., Enver T. (1998). Lymphopain, a cytotoxic T and natural killer cell-associated cysteine proteinase. Leukemia.

[45] Öörni K., Sneck M., Brömme D., Pentikäinen M.O., Lindstedt K.A., Mäyränpää M., Aitio H., Kovanen P.T. (2004). Cysteine Protease Cathepsin F Is Expressed in Human Atherosclerotic Lesions, Is Secreted by Cultured Macrophages, and Modifies Low Density Lipoprotein Particles in Vitro. J. Biol. Chem..

[46] Reddy V.Y., Zhang Q.Y., Weiss S.J. (1995). Pericellular mobilization of the tissue-destructive cysteine proteinases, cysteine proteinases B, L, and S, by human monocyte-derived macrophages. Proc. Natl. Acad. Sci. USA.

[47] Kaakinen R., Lindstedt K.A., Sneck M., Kovanen P.T., Öörni K. (2007). Angiotensin II increases expression and secretion of cathepsin F in cultured human monocyte-derived macrophages: An angiotensin II type 2 receptor-mediated effect. Atherosclerosis.

[48] Conus S., Simon H. (2010). Cathepsins and their involvement in immune responses. Swiss Med. Wkly..

[49] Shi G.-P., Bryant R.A., Riese R., Verhelst S., Driessen C., Li Z., Bromme D., Ploegh H.L., Chapman H.A. (2000). Role for Cathepsin F in Invariant Chain Processing and Major Histocompatibility Complex Class II Peptide Loading by Macrophages. J. Exp. Med..

[50] Pires D., Marques J., Pombo J.P., Carmo N., Bettencourt P., Neyrolles O., Lugo-Villarino G., Anes E. (2016). Role of Cysteine proteinases in Mycobacterium tuberculosis Survival in Human Macrophages. Sci. Rep..

[51] Kumar B.V., Connors T.J., Farber D.L. (2018). Human T Cell Development, Localization, and Function throughout Life. Immunity.

[52] Bhandoola A., Kithiganahalli B., Granger L., Singer A. (2000). Programming for cytotoxic effector function occurs concomitantly with CD4 extinction during CD8+ T cell differentiation in the thymus. Int. Immunol..

[53] Stoeckle C., Gouttefangeas C., Hammer M., Weber E., Melms A., Tolosa E. (2009). Cathepsin W expressed exclusively in CD8+ T cells and NK cells, is secreted during target cell killing but is not essential for cytotoxicity in human CTLs. Exp. Hematol..

[54] Kuester D., Vieth M., Peitz U., Kahl S., Stolte M., Roessner A., Weber E., Malfertheiner P., Wex T. (2005). Upregulation of cathepsin W-expressing T cells is specific for autoimmune atrophic gastritis compared to other types of chronic gastritis. World J. Gastroenterol..

[55] Li J., Chen Z., Kim G., Luo J., Hori S., Wu C. (2023). Cathepsin W restrains peripheral regulatory T cells for mucosal immune quiescence. Sci. Adv..

[56] Bühling F., Peitz U., Krüger S., Küster D., Vieth M., Gebert I., Roessner A., Weber E., Malfertheiner P., Wex T. (2004). Cysteine proteinases K, L, B, X and W are differentially expressed in normal and chronically inflamed gastric mucosa. Biol. Chem..

[57] Gunther S.C., Martinez-Romero C., Borau M.S., Pham C.T.N., Garcia-Sastre A., Stertz S. (2022). Proteomic Identification of Potential Target Proteins of Cathepsin W for Its Development as a Drug Target for Influenza. Microbiol. Spectr..

[58] Edinger T.O., Pohl M.O., Yángüez E., Stertz S. (2015). Cathepsin W Is Required for Escape of Influenza A Virus from Late Endosomes. mBio.

[59] Imeri J., Desterke C., Marcoux P., Chaker D., Oudrhiri N., Fund X., Faivre J., Bennaceur-Griscelli A., Turhan A.G. (2023). Case report: Long-term voluntary Tyrosine Kinase Inhibitor (TKI) discontinuation in chronic myeloid leukemia (CML): Molecular evidence of an immune surveillance. Front. Oncol..

[60] Hsu A., Podvin S., Hook V. (2018). Lysosomal Cathepsin Protease Gene Expression Profiles in the Human Brain During Normal Development. J. Mol. Neurosci..

[61] Tang C.-H., Lee J.-W., Galvez M.G., Robillard L., Mole S.E., Chapman H.A. (2006). Murine Cathepsin F Deficiency Causes Neuronal Lipofuscinosis and Late-Onset Neurological Disease. Mol. Cell. Biol..

[62] Finn R.D., Bateman A., Clements J., Coggill P., Eberhardt R.Y., Eddy S.R., Heger A., Hetherington K., Holm L., Mistry J. (2014). Pfam: The protein families database. Nucleic Acids Res..

[63] Mijanovic O., Petushkova A.I., Brankovic A., Turk B., Solovieva A.B., Nikitkina A.I., Bolevich S., Timashev P.S., Parodi A., Zamyatnin A.A. (2021). Cathepsin D—Managing the Delicate Balance. Pharmaceutics.

[64] Smith K.R., Dahl H.-H.M., Canafoglia L., Andermann E., Damiano J., Morbin M., Bruni A.C., Giaccone G., Cossette P., Saftig P. (2013). Cathepsin F variants cause Type B Kufs disease, an adult-onset neuronal ceroid lipofuscinosis. Hum. Mol. Genet..

[65] Bras J., Djaldetti R., Alves A.M., Mead S., Darwent L., Lleo A., Molinuevo J.L., Blesa R., Singleton A., Hardy J. (2016). Exome sequencing in a consanguineous family clinically diagnosed with early-onset Alzheimer’s disease identifies a homozygous CTSF mutation. Neurobiol. Aging.

[66] van der Zee J., Mariën P., Crols R., Van Mossevelde S., Dillen L., Perrone F., Engelborghs S., Verhoeven J., D’Aes T., Groote C.C.-D. (2016). Mutated *CTSF* in adult-onset neuronal ceroid lipofuscinosis and FTD. Neurol. Genet..

[67] Wang C., Xu H., Yuan Y., Lian Y., Xie N., Ming L., Wang C., Xu H., Yuan Y., Lian Y. (2018). Novel compound heterozygous variants causing Kufs disease type B. Int. J. Neurosci..

[68] Gultekin M., Tufekcioglu Z., Baydemir R. (2022). Novel frameshift CTSF variant causing kufs disease type B mimicking frontotemporal dementia-parkinsonism. Neurocase.

[69] Di Fabio R., Moro F., Pestillo L., Meschini M.C., Pezzini F., Doccini S., Casali C., Pierelli F., Simonati A., Santorelli F.M. (2014). Pseudo-dominant inheritance of a novel CTSF variant associated with type B Kufs disease. Neurology.

[70] Peters J., Rittger A., Weisner R., Knabbe J., Zunke F., Rothaug M., Damme M., Berkovic S.F., Blanz J., Saftig P. (2015). Lysosomal integral membrane protein type-2 (LIMP-2/SCARB2) is a substrate of cathepsin-F, a cysteine protease mutated in type-B-Kufs-disease. Biochem. Biophys. Res. Commun..

[71] Wang Y.-L., Zhu M.-Y., Yuan Z.-F., Ren X.-Y., Guo X.-T., Hua Y., Xu L., Zhao C.-Y., Jiang L.-H., Zhang X. (2022). Proteomic profiling of cerebrospinal fluid in pediatric myelin oligodendrocyte glycoprotein antibody-associated disease. World J. Pediatr..

[72] Hor J.Y., Fujihara K. (2023). Epidemiology of myelin oligodendrocyte glycoprotein antibody-associated disease: A review of prevalence and incidence worldwide. Front. Neurol..

[73] Takaya K., Asou T., Kishi K. (2023). Cathepsin F is a potential marker for senescent human skin fibroblasts and keratinocytes associated with skin aging. GeroScience.

[74] Julian T.H., Cooper-Knock J., MacGregor S., Guo H., Aslam T., Sanderson E., Black G.C.M., Sergouniotis P., Smith L.E.H. (2023). Phenome-wide Mendelian randomisation analysis identifies causal factors for age-related macular degeneration. eLife.

[75] Yao C., Zhou Y., Wang H., Deng F., Chen Y., Zhu X., Kong Y., Pan L., Xue L., Zhou X. (2021). Adipose-derived stem cells alleviate radiation-induced dermatitis by suppressing apoptosis and downregulating cathepsin F expression. Stem Cell Res. Ther..

[76] Korpos É., Kadri N., Kappelhoff R., Wegner J., Overall C.M., Weber E., Holmberg D., Cardell S., Sorokin L. (2013). The Peri-islet Basement Membrane, a Barrier to Infiltrating Leukocytes in Type 1 Diabetes in Mouse and Human. Diabetes.

[77] Saghizadeh M., Epifantseva I., Hemmati D.M., Ghiam C.A., Brunken W.J., Ljubimov A.V. (2013). Enhanced Wound Healing, Kinase and Stem Cell Marker Expression in Diabetic Organ-Cultured Human Corneas Upon MMP-10 and Cathepsin F Gene Silencing. Investig. Opthalmology Vis. Sci..

[78] Schechter I., Berger A. (1967). On the size of the active site in proteases. I. Papain. Biochem. Biophys. Res. Commun..

[79] Ochieng J., Chaudhuri G. (2010). Cystatin Superfamily. J. Health Care Poor Underserved.

[80] Abrahamson M., Alvarez-Fernandez M., Nathanson C.-M. (2003). Cystatins. Biochem. Soc. Symp..

[81] Fonovič M., Brömme D., Turk V., Turk B. (2004). Human cathepsin F: Expression in baculovirus system, characterization and inhibition by protein inhibitors. Biol. Chem..

[82] Langerholc T., Zavašnik-Bergant V., Turk B., Turk V., Abrahamson M., Kos J. (2005). Inhibitory properties of cystatin F and its lo-calization in U937 promonocyte cells. FEBS J..

[83] Miheliĕ M., Doberšek A., Gunĕar G., Turk D. (2008). Inhibitory fragment from the p41 form of invariant chain can regulate activity of cysteine cysteine proteinases in antigen presentation. J. Biol. Chem..

[84] Reis F.C.G., Costa T.F.R., Sulea T., Mezzetti A., Scharfstein J., Brömme D., Ménard R., Lima A.P.C.A. (2007). The propeptide of cruzipain—A potent selective inhibitor of the trypanosomal enzymes cruzipain and brucipain, and of the human enzyme cathepsin F. FEBS J..

[85] Bode W., Huber R. (2000). Structural basis of the endoproteinase–protein inhibitor interaction. Biochim. et Biophys. Acta (BBA)—Protein Struct. Mol. Enzym..

[86] Lenarcic B., Bevec T. (1998). Thyropins--new structurally related proteinase inhibitors. Biol. Chem..

[87] Rzychon M., Chmiel D., Stec-Niemczyk J. (2004). Modes of inhibition of cysteine proteases. Acta Biochim. Pol..

[88] Schmitz J., Furtmann N., Ponert M., Frizler M., Löser R., Bartz U., Bajorath J., Gütschow M. (2015). Active Site Mapping of Human Cathepsin F with Dipeptide Nitrile Inhibitors. ChemMedChem.

[89] Ho J.D., Meltser Y., Buggy J.J., Palmer J.T., Elrod K.C., Chan H., Mortara K.D., Somoza J.R. (2002). Expression, purification, crystallization and preliminary X-ray diffraction studies of human cathepsin F complexed with an irreversible vinyl sulfone inhibitor. Acta Crystallogr. Sect. D Struct. Biol..

[90] Somoza J.R., Palmer J.T., Ho J.D. (2002). The Crystal Structure of Human Cathepsin F and Its Implications for the Development of Novel Immunomodulators. J. Mol. Biol..

[91] Falgueyret J.-P., Black W.C., Cromlish W., Desmarais S., Lamontagne S., Mellon C., Riendeau D., Rodan S., Tawa P., Wesolowski G. (2004). An activity-based probe for the determination of cysteine cathepsin protease activities in whole cells. Anal. Biochem..

[92] Rozman-Pungerčar J., Kopitar-Jerala N., Bogyo M., Turk D., Vasiljeva O., Štefe I., Vandenabeele P., Brömme D., Puizdar V., Fonović M. (2003). Inhibition of papain-like cysteine proteases and legumain by caspase-specific inhibitors: When reaction mechanism is more important than specificity. Cell Death Differ..

[93] Rudzińska M., Parodi A., Maslova V.D., Efremov Y.M., Gorokhovets N.V., Makarov V.A., Popkov V.A., Golovin A.V., Zernii E.Y., Zamyatnin A.A. (2020). Cysteine Cysteine proteinases Inhibition Affects Their Expression and Human Renal Cancer Cell Phenotype. Cancers.

